# Characterization and Comparative Genomic Analyses of *Pseudomonas aeruginosa* Phage PaoP5: New Members Assigned to PAK_P1-like Viruses

**DOI:** 10.1038/srep34067

**Published:** 2016-09-23

**Authors:** Mengyu Shen, Shuai Le, Xiaolin Jin, Gang Li, Yinling Tan, Ming Li, Xia Zhao, Wei Shen, Yuhui Yang, Jing Wang, Hongbin Zhu, Shu Li, Xiancai Rao, Fuquan Hu, Shuguang Lu

**Affiliations:** 1 Department of Microbiology, College of Basic Medical Science, Third Military Medical University, Chongqing, 400038, China

## Abstract

As a potential alternative to antibiotics, phages can be used to treat multi-drug resistant bacteria. As such, the biological characteristics of phages should be investigated to utilize them as effective antimicrobial agents. In this study, phage PaoP5, a lytic virus that infects *Pseudomonas aeruginosa* PAO1, was isolated and genomically characterized. PaoP5 comprises an icosahedral head with an apex diameter of 69 nm and a contractile tail with a length of 120 nm. The PaoP5 genome is a linear dsDNA molecule containing 93,464 base pairs (bp) with 49.51% G + C content of 11 tRNA genes and a 1,200 bp terminal redundancy. A total of 176 protein-coding genes were predicted in the PaoP5 genome. Nine PaoP5 structural proteins were identified. Three hypothetical proteins were determined as structural. Comparative genomic analyses revealed that seven new *Pseudomonas* phages, namely, PaoP5, K8, C11, vB_PaeM_C2-10_Ab02, vB_PaeM_C2-10_Ab08, vB_PaeM_C2-10_Ab10, and vB_PaeM_C2-10_Ab15, were similar to PAK_P1-like viruses. Phylogenetic and pan-genome analyses suggested that the new phages should be assigned to PAK_P1-like viruses, which possess approximately 100 core genes and 150 accessory genes. This work presents a detailed and comparative analysis of PaoP5 to enhance our understanding of phage biology.

Bacteriophages or phages are abundant viruses that infect bacteria. The number of phages is approximately 10-fold higher than that of bacteria^1^. Since their discovery in 1915, phages have influenced basic and applied biology^2^. Since 1959, nearly 6,300 different phages have been examined through electron microscopy, including 6,196 bacterial and 88 archaeal phages^3^. In October 2012, 759 phages, including 721 infecting bacteria and 38 infecting archaea, were completely sequenced^4^. In February 2016, the number of completely sequenced phages reached 2,012, including 1,935 infecting bacteria and 77 infecting archaea, as revealed by the data from the National Center for Biotechnology Information (Bethesda, MA, USA). This number is lower than that of completely sequenced bacteria, which reached 5,020 in February 2016, although the genome size of phages is less than that of bacteria. Novel phages should be characterized and genomically analyzed to obtain additional valuable data regarding phages and help enhance our understanding of the evolutionary relationships between phages and bacteria.

As a Gram-negative opportunistic pathogen, *Pseudomonas aeruginosa* is the leading cause of local and systemic nosocomial infections; in some cases, its infection is life threatening^5^. *P*. *aeruginosa* infections are difficult to treat with antibiotics because of its intrinsic multi-drug resistance^6^. Thus, the biological characteristics of *P*. *aeruginosa* phages should be investigated to eradicate this notorious pathogen^7^. *P*. *aeruginosa* phages are taxonomically diverse and genetically dissimilar; they have been widely considered for their application as therapeutic and typing agents^8^. As of February 2016, 141 complete genome sequences of *Pseudomonas* phages mostly infecting *P*. *aeruginosa* have become available in GenBank^9^. *P*. *aeruginosa* phages are classified into several distinct genera, namely, PAK_P1-like^10^, KPP10-like^11^, and PB1-like viruses^12^. With the rapid development of genome sequencing, numerous novel *P*. *aeruginosa* phages have been identified. However, most of these phages have remained unclassified. Therefore, novel *P*. *aeruginosa* phages should be characterized and classified to facilitate the understanding of the interactions between *P*. *aeruginosa* and its phages and to help develop new approaches that combat this versatile pathogen.

## Results and Discussion

### Biological features of PaoP5

**Figure 1:**
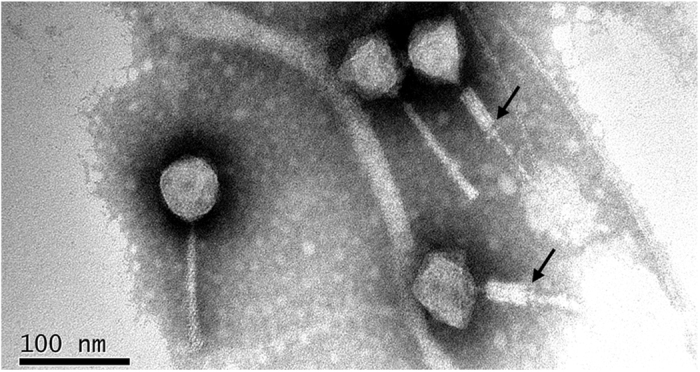
Electron micrograph of PaoP5 phage particles. The sample was stained with phosphotungstate. The scale bar represents 100 nm. The black arrows indicate contracted tails.

Phage PaoP5 was isolated from hospital sewage using *P*. *aeruginosa* PAO1 as host bacterium. PaoP5 was cultured overnight (~12 h) and formed large, clear plaques (~5 mm in diameter) on double agar plates. This finding suggested that PaoP5 is a lytic phage. Transmission electron microscopy analysis indicated that the head structure of PaoP5 is an icosahedron with an apex diameter of approximately 69 nm (Fig. 1). The non-contracted tail is about 120 nm in length. The contracted tail consists of a central tube, a 55 nm long contracted sheath and an 8 nm long neck. The morphological characteristics of phage PaoP5 suggest its membership under the *Myoviridae* family, members of which can affect many aspects of bacterial ecology and are efficient killers of bacteria, making them suitable for phage therapy^13^. Several attempts were made to explore the anti-bacterial potential of PAK_P1-like viruses^14^,^15^,^16^. New *P*. *aeruginosa* phages are continuously isolated, and their capacity to target various clinical strains need to be tested *in vitro* and in animal models.

### Genomic characteristics of PaoP5

The length of the PaoP5 genome sequence is 93,464 bp, with an average G + C content of 49.51%, which is significantly less than that of its bacterial host (66.35%). The general features of the PaoP5 genome are listed in Table S1. Genome termini analysis revealed that PaoP5 holds a direct terminal repeat (DTR) with a length of approximately 1,200 bp (Fig. S1). The PaoP5 genome can be divided into six functional modules, of which two functionally unknown modules situate near the 5′ and 3′ ends of the PaoP5 genome, respectively, and many small genes with unknown functions cluster in the two modules (Fig. S2). In addition, among the 176 predicted proteins of PaoP5, only 19.3% hold putative functions. Therefore, the vast number of phage genes with unknown functions should be explored extensively to better understand this interesting virus. The mosaic genome structure of PaoP5 suggests that its genome sequence may be evolved from combinations of modules from different species, similar to other tailed phage genomes^17^. The complete genome sequence and annotations of PaoP5 have been deposited in GenBank under the accession number KU297675.

### Identification of phage PaoP5 structural proteins

**Figure 2:**
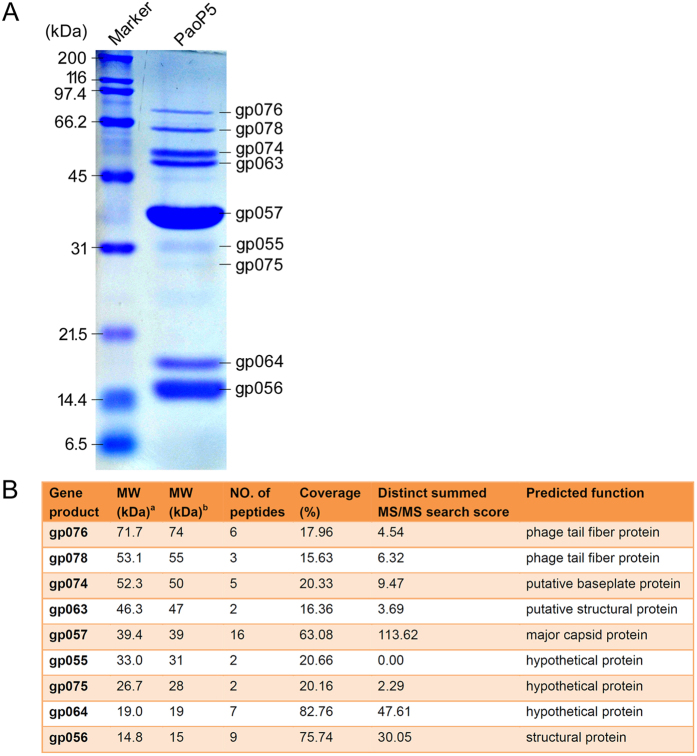
Identification of PaoP5 structural proteins. (**A**) SDS–PAGE analysis. Proteins were visualized in a 15% (w/v) gel. (**B**) Detailed results of HPLC–MS analysis. ^a^The MW value was theoretically calculated. ^b^The MW value was experimentally estimated.

To identify the structural proteins of PaoP5, sodium dodecyl sulfate–polyacrylamide gel electrophoresis (SDS–PAGE) was used to separate and visualize each structural protein in the gel (Fig. 2A). Nine proteins with molecular weights ranging from 15 kDa to 76 kDa were determined. Then, each protein band was excised for high-performance liquid chromatography (HPLC)–mass spectrometry (MS), permitting the allocation of nine protein bands to nine corresponding PaoP5 genes. The detailed parameters and results of mass spectrometry are shown in Fig. 2B. The lowest sequence coverage was 15.63% for gp078, but the MS search score was not the lowest. Notably, the MS search score of gp055 was 0.00, indicating that the score should be further verified. Three hypothetical proteins, including gp055, gp064, and gp075 (Fig. 2B), were separated by SDS–PAGE, suggesting that these are actually structural proteins. Thus, we updated the corresponding GenBank records of gp055, gp064 and gp075, and conferred them with the function “structural protein” instead of “hypothetical protein.” As expected, the predominant band was predicted as the major capsid protein (gp057, ~39 kDa) of PaoP5. The structural proteins with molecular weight higher than 35 kDa, including tail fiber, baseplate and major capsid, are important for phage PaoP5 morphogenesis.

### New members (including PaoP5) were assigned to PAK_P1-like viruses

BlastN analysis revealed that the genome sequences of phages PaP1^4^,^18^,^19^,^20^, K8, C11, JG004^21^, vB_PaeM_C2-10_Ab1^14^,^22^, vB_PaeM_C2-10_Ab02^22^, vB_PaeM_C2-10_Ab08^22^, vB_PaeM_C2-10_Ab10^22^, vB_PaeM_C2-10_Ab15^22^, PAK_P1^10^,^15^,^16^, PAK_P2^10^,^16^, and PAK_P4^10^,^16^ share an identity of above 90% and query coverage also above 90% with the PaoP5 genome (Table S2 and Fig. S3). These similar phages were isolated in different areas around the world, spanning Asia, Europe and Africa (Fig. S4), suggesting the complex evolutionary relationships among these phages. The tBlastX analysis of the 13 phage genomes revealed that these phages show striking similarities at the protein level (Fig. S5).

**Figure 3:**
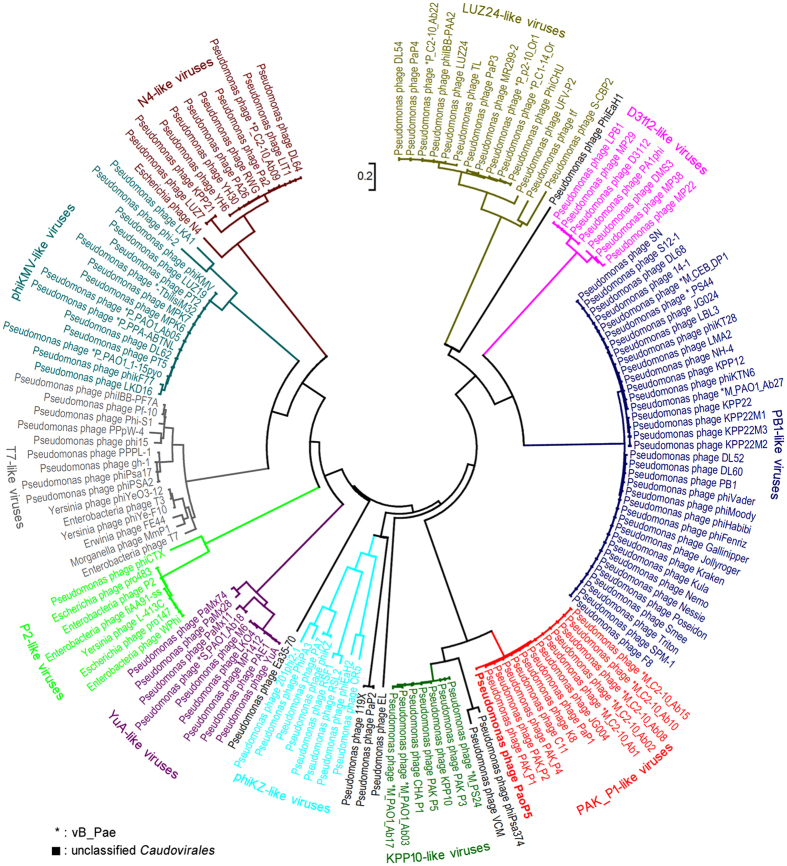
Phylogenetic relationships of *Caudovirales* infecting *Pseudomonas* species. Major capsid proteins and the neighbor-joining method were used to construct the phylogenetic tree. Different clades are marked with different colors. PAK_P1-like viruses are marked in red. The scale length of relative evolution distance is 0.2.

As of February 3, 2016, 1,718 complete genomes of *Caudovirales*, including 463 *Myoviridaes*, 319 *Podoviridaes*, 913 *Siphoviridaes*, and 23 unclassified *Caudovirales* were released in GenBank^9^. Among the 1,718 *Caudovirales*, 134 members infect *Pseudomonas* species (mostly *P*. *aeruginosa*). We conducted a phylogenetic analysis of 149 phages, including 134 members of *Caudovirales* that infect *Pseudomonas* species and 15 related members of *Caudovirales* that infect bacteria of other genera. The result indicated that the phages of the same genus are clustered into one clade, making the phylogenetic tree divide into several clades (Fig. 3). As expected, PaoP5 was clustered into PAK_P1-like viruses. In the year 2015, PAK_P1-like viruses was reported to have six members, including PaP1, JG004, vB_PaeM_C2-10_Ab1, PAK_P1, PAK_P2, and PAK_P4^10^. Herein, we proposed that seven new members, including PaoP5, K8, C11, vB_PaeM_C2-10_Ab02, vB_PaeM_C2-10_Ab08, vB_PaeM_C2-10_Ab10, and vB_PaeM_C2-10_Ab15, should be assigned to PAK_P1-like viruses (Fig. 3). Although distributed in different clades, phages VCM, phiPsa374, and KPP10-like viruses are closely related to PAK_P1-like viruses (Fig. 3). Thus, all of these phages should be grouped into the subfamily of the *Myoviridae* named *Felixounavirinae* as proposed previously^10^.

Given the 13 complete genomes of PAK_P1-like viruses, we performed a pan-genome analysis. Results showed that the pan-genome of PAK_P1-like viruses comprises approximately 100 core genes and 150 accessory genes (Fig. S6). Hence, new additional members of PAK_P1-like viruses are predicted to be characterized and sequenced in the near future. The core genome of this phage genus is expected to contain less than 100 genes, but infinitely close to a certain amount, which constitutes the minimal genome^23^ of these phages, thus providing useful clues for synthetic biology analysis^24^.

## Materials and Methods

### Bacterial strains and culture condition

*P*. *aeruginosa* PAO1^18^,^21^ was used as the host bacterium of phage PaoP5. As for host spectrum assay of PaoP5, the tested 95 *P*. *aeruginosa* strains were isolated from the Department of Burn of the first affiliated hospital of the Third Military Medical University (Southwest Hospital, Chongqing, China) and cultivated in our laboratory. Bacteria were grown in Luria–Bertani (LB) liquid medium or plated onto solid LB medium containing 1.5% (w/v) agar and cultured at 37 °C with aeration.

### Transmission electron microscopy (TEM)

Filtered phage lysates (~10^11 ^PFU/mL) were placed on copper grids to allow adsorption for 10 min, then negatively stained with 2% phosphotungstic acid (PT-A, pH 4.5) for 1 min and subsequently air dried. Phage particles were observed using TECNAI 10 electron microscope (Philips, The Netherlands) at a voltage of 80 kV and with a magnification of 130,000. Images were acquired digitally with a camera (gatan Model 785) inside the microscope. Brightness and contrast were adjusted with Adobe Photoshop CS5.

### SDS–PAGE and HPLC–MS of the PaoP5 structural proteins

Structural protein analysis was performed as described previously^4^. Briefly, the purified phage particles were heat-denaturized and loaded onto a 15% (w/v) polyacrylamide gel to visualize PaoP5 structural proteins. SDS–PAGE (12% [w/v] and 10% [w/v]) was also performed to better separate proteins with different molecular weights. Proteins were stained with Coomassie Brilliant Blue R250 dye and washed with methanol–acetic acid–H_2_O. Then, protein bands were excised from the gel for HPLC–MS analysis. The data from HPLC–MS analysis were processed by Agilent Spectrum Mill proteomics software to allocate each band to the corresponding gene.

### Comparative genomic analysis

Thirteen complete phage genome sequences were subjected to BlastN comparisons by using blast 2.2.29+ (ftp://ftp.ncbi.nlm.nih.gov/blast/)^25^,^26^ and visualized by BRIG (http://brig.sourceforge.net/)^27^ with a 80% identity cut-off. The PaoP5 genome sequence was used as reference. Phage genome sequences were subjected to tBlastX analysis by using EasyFig (http://mjsull.github.io/Easyfig/)^28^ with a 33% identity cut-off. Major capsid protein sequences of phages (belonging to *Caudovirales*) infecting *P*. *aeruginosa* were downloaded from GenBank^9^. The multiple sequence alignments of major capsid protein sequences were conducted using ClustalW^29^ with default parameters, and the phylogenetic tree was constructed and displayed by MEGA 6.06 (http://www.megasoftware.net/)^30^ with the neighbor-joining method^31^. We then constructed a Venn diagram using an online tool for calculating and drawing custom Venn diagrams (http://bioinformatics.psb.ugent.be/webtools/Venn/). Pan-genome analyses were performed using CoreGenes 3.5 Batch Submission Tool (http://binf.gmu.edu:8080/CoreGenes3.5/BatchCoreGenes.html)^32^ and Panseq (https://lfz.corefacility.ca/panseq/analyses#userPan)^33^ with default parameters, and the results of CoreGenes and Panseq were combined to better present the pan-genome of PAK_P1-like viruses.

## Additional InformationAccession code

**Accession code:** The complete genome sequence and annotations of PaoP5 have been deposited in GenBank under the accession number KU297675.

**How to cite this article**: Shen, M. *et al*. Characterization and Comparative Genomic Analyses of *Pseudomonas aeruginosa* Phage PaoP5: New Members Assigned to PAK_P1-like Viruses. *Sci. Rep.* **6**, 34067; doi: 10.1038/srep34067 (2016).

## Supplementary Material

Supplementary Information
