# Supplementary material for: Characterization and Comparative Genomic Analyses of Pseudomonas aeruginosa Phage PaoP5: New Members Assigned to PAK_P1-like Viruses
Source: Sci Rep. 2016 Sep 23;6:34067. doi: 10.1038/srep34067 (PMC5034324; doi:10.1038/srep34067)
Supplement: Supplementary Information [file srep34067-s1.pdf]

Characterization and Comparative Genomic Analyses of *Pseudomonas aeruginosa* Phage PaoP5: New Members Assigned to PAK_P1-like Viruses

Mengyu Shen, Shuai Le, Xiaolin Jin, Gang Li, Yinling Tan, Ming Li, Xia Zhao, Wei Shen, Yuhui Yang, Jing Wang, Hongbin Zhu, Shu Li, Xiancai Rao, Fuquan Hu & Shuguang Lu

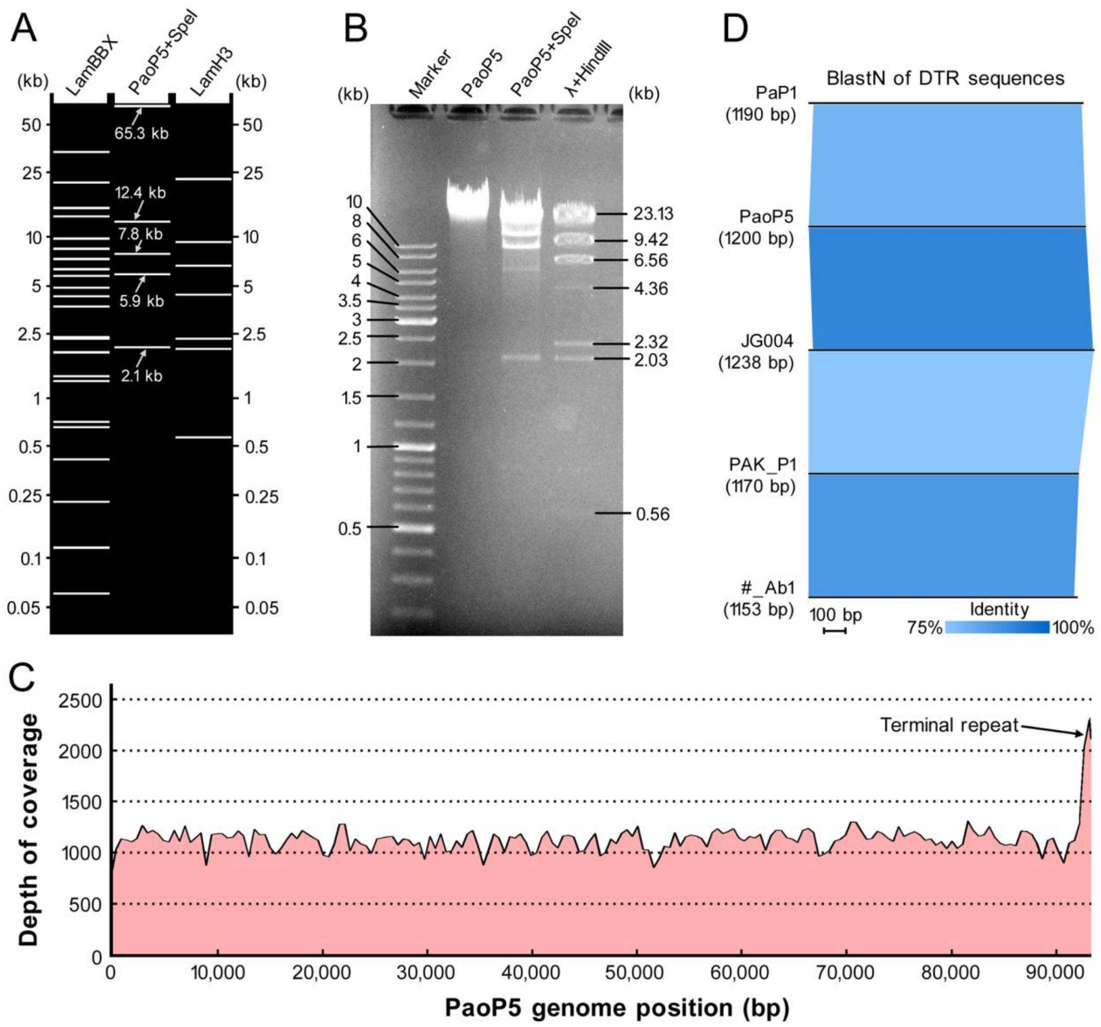

Figure S1. Analysis of PaoP5 genome ends. (**A**) Agarose gel simulation for digestion of PaoP5 genome DNA by restriction endonuclease SpeI. The length of each band is indicated by arrows. (**B**) Digestion of the PaoP5 genome DNA by SpeI. (**C**) Sequencing coverage of the PaoP5 genome, revealing a terminal repeat. (**D**) BlastN comparison of the DTR sequences of phages PaoP5, PaP1, JG004, PAK_P1, and vB_PaeM_C2-10_Ab1. # represents vB_PaeM_C2-10. The lengths of the corresponding DTR sequences are indicated in the parentheses.

Characterization and Comparative Genomic Analyses of *Pseudomonas aeruginosa* Phage PaoP5: New Members Assigned to PAK_P1-like Viruses

Mengyu Shen, Shuai Le, Xiaolin Jin, Gang Li, Yinling Tan, Ming Li, Xia Zhao, Wei Shen, Yuhui Yang, Jing Wang, Hongbin Zhu, Shu Li, Xiancai Rao, Fuquan Hu & Shuguang Lu

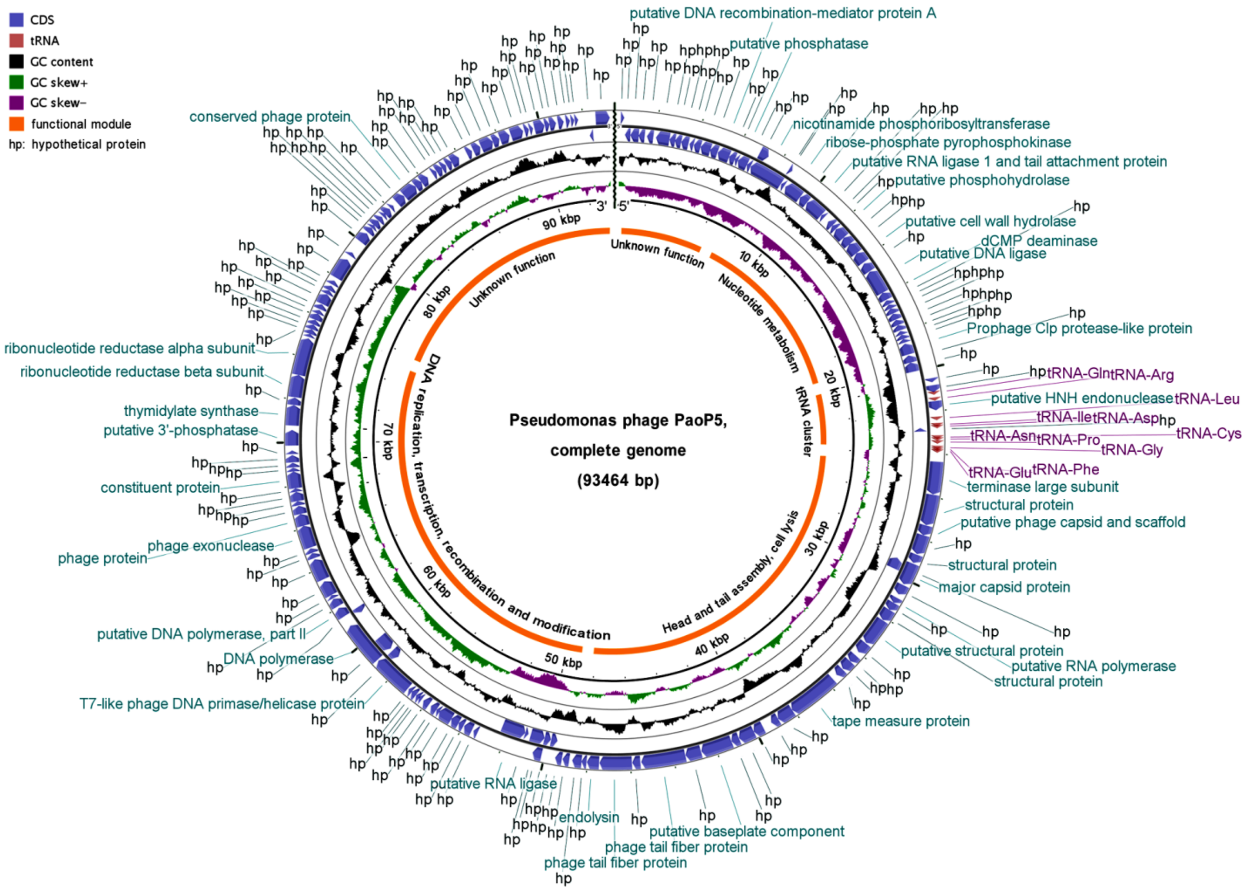

Figure S2. Circular presentation of the PaoP5 genome. The names of the predicted genes (also shown in the GenBank file of PaoP5 genome) are indicated in the outermost region. The outermost ring denotes genes on the plus strand, followed by rings that depict genes on the minus strand, GC content (black), GC skew (purple/green), position scales and functional modules, respectively.

Characterization and Comparative Genomic Analyses of *Pseudomonas aeruginosa* Phage PaoP5: New Members Assigned to PAK_P1-like Viruses

Mengyu Shen, Shuai Le, Xiaolin Jin, Gang Li, Yinling Tan, Ming Li, Xia Zhao, Wei Shen, Yuhui Yang, Jing Wang, Hongbin Zhu, Shu Li, Xiancai Rao, Fuquan Hu & Shuguang Lu

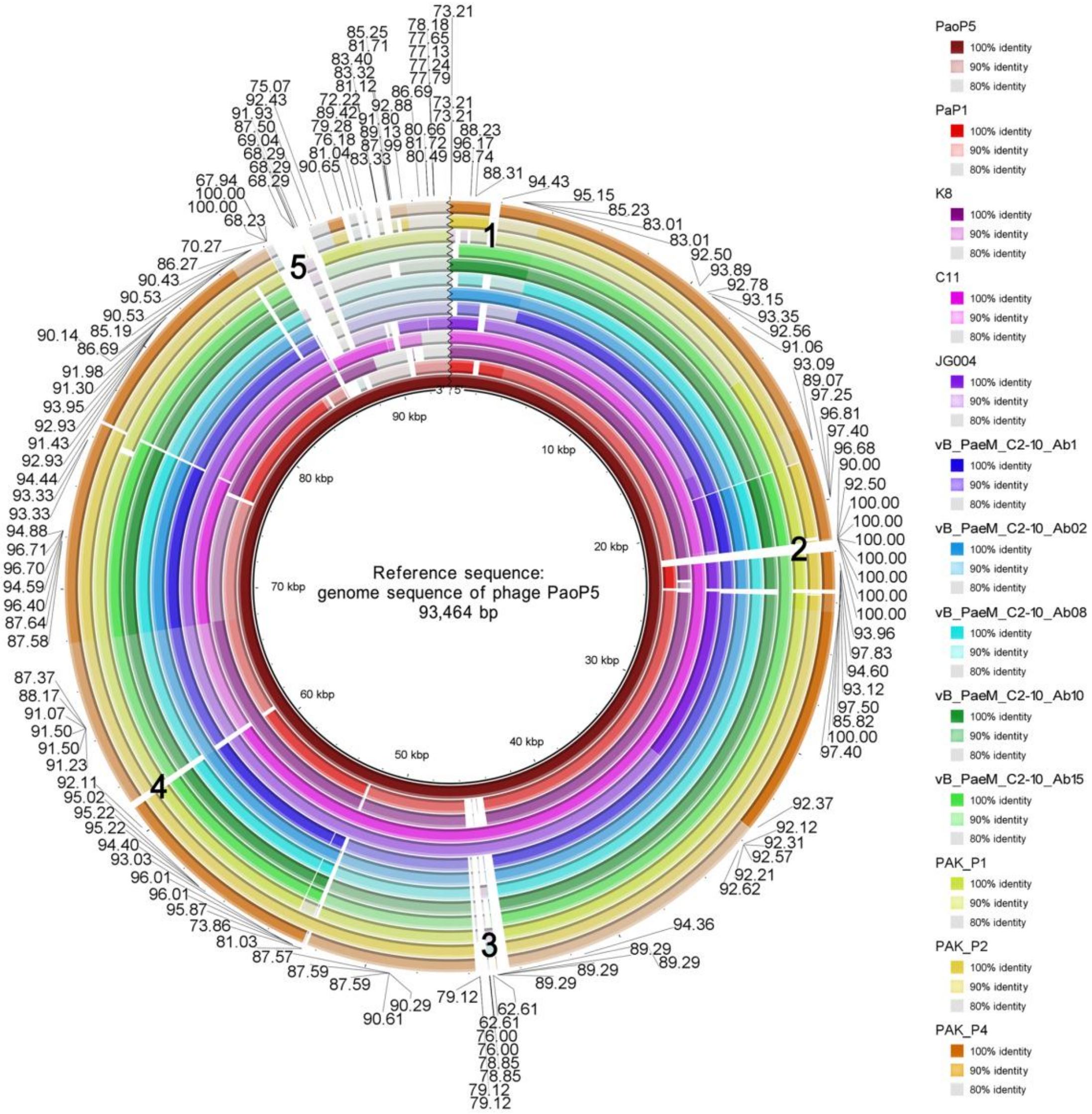

Figure S3. BRIG comparison of complete genome sequences of PaoP5 with other 12 phages listed in Table S2. The BlastN method was employed to perform the comparison. Identity labels of the 13 phage genomes are shown in the same order as

the rings from the innermost to the outermost. Identities of the corresponding comparison fragments are shown in the outermost region of the figure. Large low-identity genomic regions (over 270 bp in length) are indicated by the numbers from 1 to 5. The lengths of these five regions are 386, 470, 1,322, 272, and 1,900 bp, respectively. Region 1 is contained in a hypothetical protein and region 5 contains 4 hypothetical proteins. Regions 2, 3, and 4 are contained in a putative HNH endonuclease, a tail fiber protein and a DNA polymerase, respectively. These five regions are probably highly variable in the process of phage evolution, and some of these regions could be exchanged by horizontal gene transfer (HGT).

Characterization and Comparative Genomic Analyses of *Pseudomonas aeruginosa* Phage PaoP5: New Members Assigned to PAK_P1-like Viruses

Mengyu Shen, Shuai Le, Xiaolin Jin, Gang Li, Yinling Tan, Ming Li, Xia Zhao, Wei Shen, Yuhui Yang, Jing Wang, Hongbin Zhu, Shu Li, Xiancai Rao, Fuquan Hu & Shuguang Lu

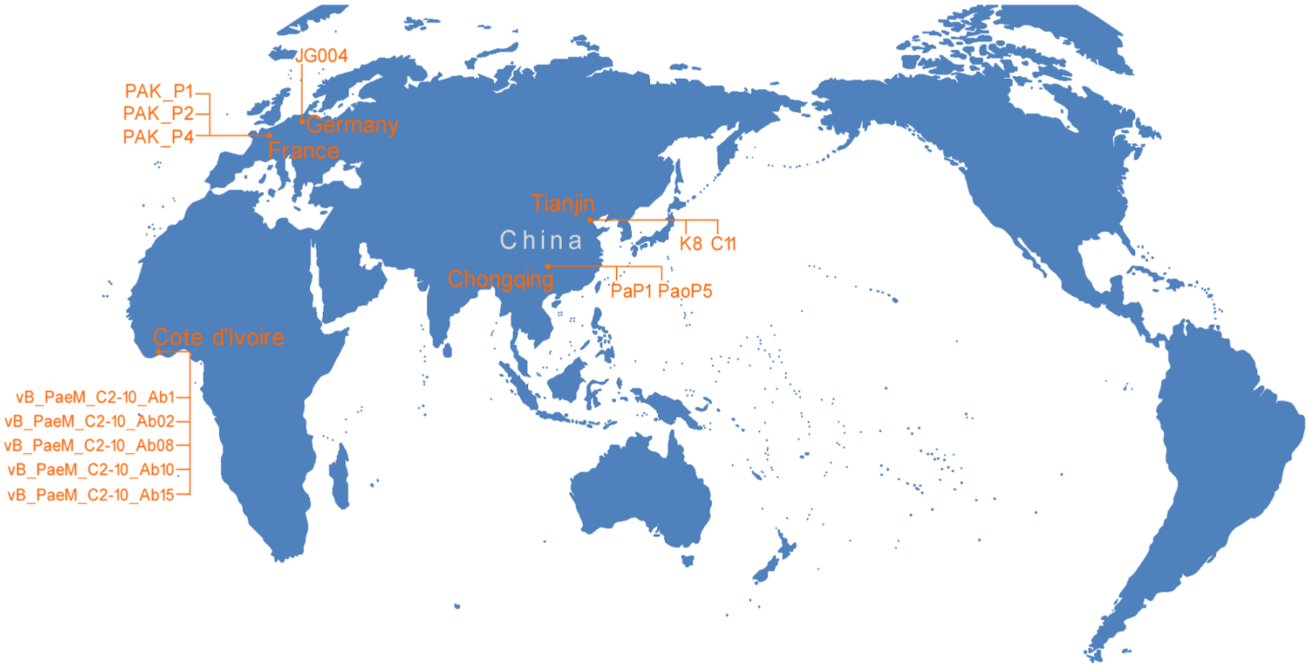

Figure S4. Geographical distribution of PAK_P1-like viruses. The diagram of world map was generated by software OfficeAssist ver. 0239.80.12012 (http://meihua.docer.com/). The text labels were added using Microsoft PowerPoint ver. 14.0.4760.1000 (https://www.microsoft.com/zh-cn).

Characterization and Comparative Genomic Analyses of *Pseudomonas aeruginosa* Phage PaoP5: New Members Assigned to PAK_P1-like Viruses

Mengyu Shen, Shuai Le, Xiaolin Jin, Gang Li, Yinling Tan, Ming Li, Xia Zhao, Wei Shen, Yuhui Yang, Jing Wang, Hongbin Zhu, Shu Li, Xiancai Rao, Fuquan Hu & Shuguang Lu

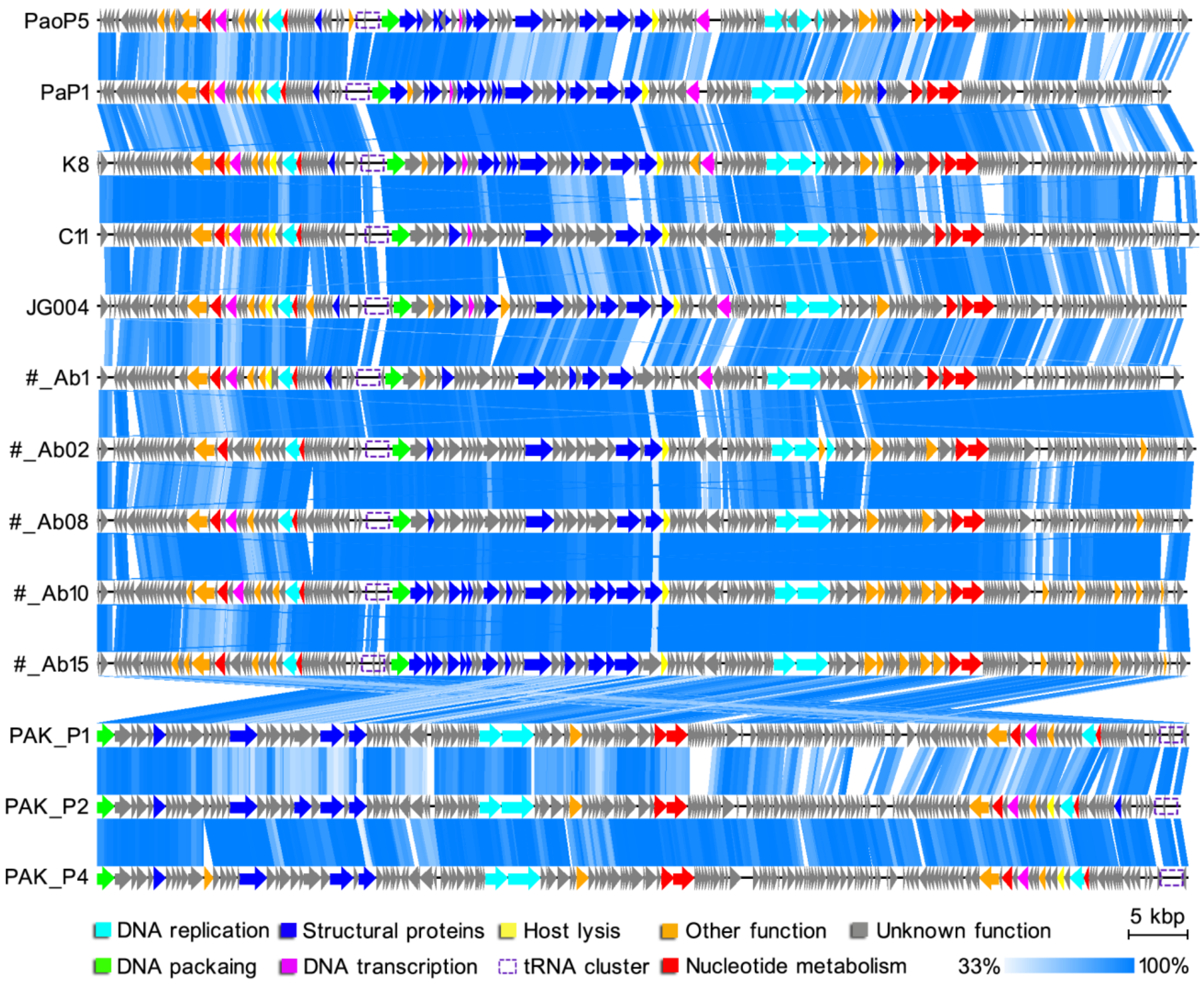

Figure S5. Visualized tBlastX comparison of the 13 phage genomes. Arrowheads denote genes, and the identity cut-off is set as 33%. # represents vB_PaeM_C2-10. Genome annotations for vB_PaeM_C2-10_Ab10 and vB_PaeM_C2-10_Ab15 were performed in this study. Other function means DNA recombination-mediator protein, phosphatase, nicotinamide phosphoribosyltransferase, phosphohydrolase,

phosphoesterase, pyrophosphatase, HNH endonuclease, phage exonuclease, conserved phage protein, ATPase, DNA methyltransferase, or putative autotransporter.

Characterization and Comparative Genomic Analyses of *Pseudomonas aeruginosa* Phage PaoP5: New Members Assigned to PAK_P1-like Viruses

Mengyu Shen, Shuai Le, Xiaolin Jin, Gang Li, Yinling Tan, Ming Li, Xia Zhao, Wei Shen, Yuhui Yang, Jing Wang, Hongbin Zhu, Shu Li, Xiancai Rao, Fuquan Hu & Shuguang Lu

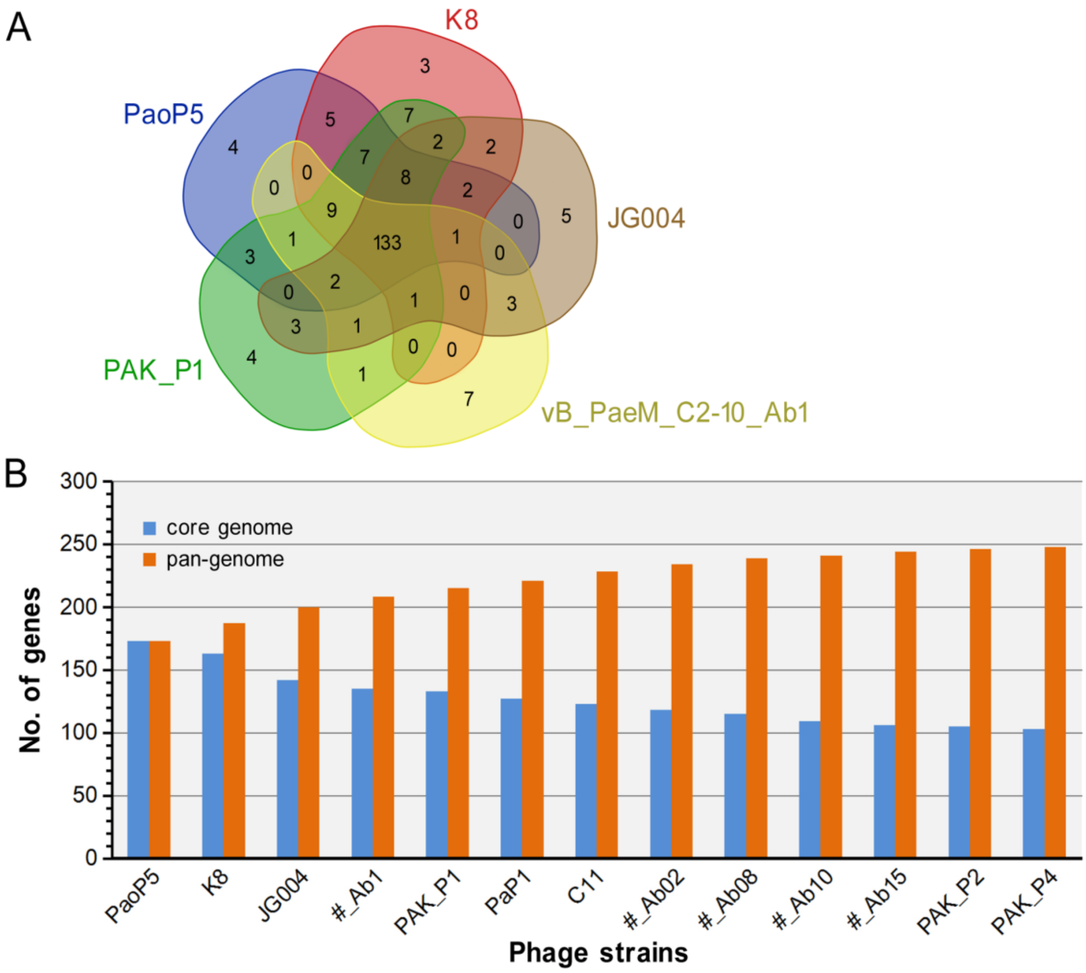

Figure S6. Sizes of the pan-genome and core genome of PAK_P1-like viruses. (**A**) Venn diagram of genomes of five typical members of PAK_P1-like viruses. (**B**) The pan-genome and core genome of PAK_P1-like viruses. # represents vB_PaeM_C2-10.

Characterization and Comparative Genomic Analyses of *Pseudomonas aeruginosa* Phage PaoP5: New Members Assigned to PAK_P1-like Viruses

Mengyu Shen, Shuai Le, Xiaolin Jin, Gang Li, Yinling Tan, Ming Li, Xia Zhao, Wei Shen, Yuhui Yang, Jing Wang, Hongbin Zhu, Shu Li, Xiancai Rao, Fuquan Hu & Shuguang Lu

Table S1. General features of the PaoP5 genome.

Features	PaoP5 genome
Genome size	93,464 bp
G+C content (G+C content host)	49.51% (66.35%)
No. of predicted genes (proteins)	187 (176)
Average length of protein-coding sequences	470 (bp)
% of the genome with non-coding regions	11.52%
No. of proteins without homologs	13
No. of proteins with predicted functions	34
No. of frameshifted Genes	78
Predicted tRNAs	$tRNA^{Glu}$; $tRNA^{Phe}$; $tRNA^{Gly}$; $tRNA^{Pro}$; $tRNA^{Asn}$; $tRNA^{Cys}$; $tRNA^{Asp}$; $tRNA^{Ile}$; $tRNA^{Leu}$; $tRNA^{Arg}$; $tRNA^{Gln}$

Characterization and Comparative Genomic Analyses of *Pseudomonas aeruginosa* Phage PaoP5: New Members Assigned to PAK_P1-like Viruses

Mengyu Shen, Shuai Le, Xiaolin Jin, Gang Li, Yinling Tan, Ming Li, Xia Zhao, Wei Shen, Yuhui Yang, Jing Wang, Hongbin Zhu, Shu Li, Xiancai Rao, Fuquan Hu & Shuguang Lu

Table S2. Comparison of phages with BlastN query coverage above 90% of the PaoP5 genome.

Phage	Isolated place	Accession	Genome size (bp)	% GC	Proteins (n)	tRNAs (n)	Query coverage (%)	Identity (%)	Ref.
PaP1	China	HQ832595	91,715	49.36	167	12	94	92	[1-4]
K8	China	KT736033	93,879	49.36	178	13	95	93	-
C11	China	KT804923	94,109	49.39	172	12	98	94	-
JG004	Germany	GU988610	93,017	49.26	161	12	96	90	[5]
#_Ab1	Cote d'Ivoire	HE983845	92,777	49.28	158	11	94	92	[6,7]
#_Ab02	Cote d'Ivoire	LN6105729	93,848	49.40	175	14	95	93	[7]
#_Ab08	Cote d'Ivoire	LN610575	93,503,	49.21	175	14	95	93	[7]
#_Ab10*	Cote d'Ivoire	LN610586	93,053	49.25	176	11	95	92	[7]
#_Ab15*	Cote d'Ivoire	LN610587	93,308	49.35	177	12	95	92	[7]
PAK_P1	France	KC862297	93,198	49.50	181	13	94	95	[8-10]
PAK_P2	France	KC862298	92,495	49.29	176	11	93	93	[9,10]

PAK_P4	France	KC862300	93,147	49.27	174	13	93	93	[9,10]

# represents vB_PaeM_C2-10.

*The genome annotations of the two phages were done using RAST[11,12] (Rapid Annotation using Subsystem Technology) in this study.

References

1 Lu, S. *et al.* Genomic and proteomic analyses of the terminally redundant genome of the Pseudomonas aeruginosa phage PaP1: establishment of genus PaP1-like phages. *Plos One* **8**, e62933, doi:10.1371/journal.pone.0062933 (2013).

2 Le, S. *et al.* Mapping the Tail Fiber as the Receptor Binding Protein Responsible for Differential Host Specificity of Pseudomonas aeruginosa Bacteriophages PaP1 and JG004. *Plos One* **8**, e68562, doi:10.1371/journal.pone.0068562 (2013).

3 Le, S. *et al.* Chromosomal DNA deletion confers phage resistance to Pseudomonas aeruginosa. *Scientific reports* **4**, 4738, doi:10.1038/srep04738 (2014).

4 Lu, S. *et al.* Unlocking the mystery of the hard-to-sequence phage genome: PaP1 methylome and bacterial immunity. *Bmc Genomics* **15**, 803, doi:10.1186/1471-2164-15-803 (2014).

5 Garbe, J., Bunk, B., Rohde, M. & Schobert, M. Sequencing and Characterization of Pseudomonas aeruginosa phage JG004. *BMC microbiology* **11**, 102, doi:10.1186/1471-2180-11-102 (2011).

6 Essoh, C. *et al.* The Susceptibility of Pseudomonas aeruginosa Strains from Cystic Fibrosis Patients to Bacteriophages. *Plos One* **8**, e60575, doi:10.1371/journal.pone.0060575 (2013).

7 Essoh, C. *et al.* Investigation of a Large Collection of Pseudomonas aeruginosa Bacteriophages Collected from a Single Environmental Source in Abidjan, Cote d'Ivoire. *Plos One* **10**, e0130548, doi:10.1371/journal.pone.0130548 (2015).

8 Debarbieux, L. *et al.* Bacteriophages can treat and prevent Pseudomonas aeruginosa lung infections. *The Journal of infectious diseases* **201**, 1096-1104, doi:10.1086/651135 (2010).

9 Henry, M., Lavigne, R. & Debarbieux, L. Predicting In Vivo Efficacy of Therapeutic Bacteriophages Used To Treat Pulmonary Infections. *Antimicrobial agents and chemotherapy* **57**, 5961-5968, doi:10.1128/Aac.01596-13 (2013).

10 Henry, M. *et al.* The search for therapeutic bacteriophages uncovers one new subfamily and two new genera of Pseudomonas-infecting Myoviridae. *Plos One* **10**, e0117163, doi:10.1371/journal.pone.0117163 (2015).

11 Overbeek, R. *et al.* The SEED and the Rapid Annotation of microbial genomes using Subsystems Technology (RAST). *Nucleic Acids Res* **42**, D206-214, doi:10.1093/nar/gkt1226 (2014).

12 Brettin, T. *et al.* RASTtk: a modular and extensible implementation of the RAST algorithm for building custom annotation pipelines and annotating batches of genomes. *Scientific reports* **5**, 8365, doi:10.1038/srep08365 (2015).

Characterization and Comparative Genomic Analyses of *Pseudomonas aeruginosa* Phage PaoP5: New Members Assigned to PAK_P1-like Viruses

Mengyu Shen, Shuai Le, Xiaolin Jin, Gang Li, Yinling Tan, Ming Li, Xia Zhao, Wei Shen, Yuhui Yang, Jing Wang, Hongbin Zhu, Shu Li, Xiancai Rao, Fuquan Hu & Shuguang Lu

Text S1. Supplementary Materials and Methods.

Phage isolation, propagation, and purification. Phage PaoP5 was isolated from the sewage of Southwest Hospital (Chongqing, China) given a standard lambda phage isolation protocol[1]. Briefly, sewage samples were centrifuged at 10,000 *g* for 10 min. Subsequently, the supernatant was filtered through a 0.22 μm filter. Then, 3 mL PAO1 culture at log phase was added to the supernatant and cultured at 37 ℃ for 12 h. The culture was filtered again through a 0.22 μm filter to remove bacteria. The supernatant was serially diluted and spotted on the bacterial lawn according to the double-agar layer method. After incubation at 37 ℃ overnight, a single plaque was separated, and phages were obtained and added to a culture of PAO1, which was at the log phase. After incubation at 37 ℃ for 6 h, a few drops of chloroform were added to the culture. The culture was then centrifuged for 5 min at 10,000 *g* and filtered through a 0.22 μm filter. The resulting phage lysate was stored at 4 ℃ for further analysis. Crude phage suspensions of PaoP5 were concentrated and purified by

PEG8000 precipitation in accordance with the method of Govind *et al.*[2]. The purified PaoP5 particles were further purified by CsCl gradient ultracentrifugation[3].

DNA sequencing. DNA extraction and purification were performed as described previously[4]. Briefly, PaoP5 particles purified by CsCl gradient ultracentrifugation were digested by proteinase K (50 μg mL^{-1}) at 56 ℃ for 1 h to release phage DNA. Then, the mixture was purified with phenol–chloroform–isoamyl alcohol (25:24:1) at 5,000 *g* for 10 min. The aqueous layer was extracted again with chloroform at 5, 000 *g* for 10 min. Isopropanol was used to precipitate DNA, and the precipitated DNA was collected and washed with ethanol. The obtained PaoP5 DNA was suspended in TE buffer (pH 8.0) for use. DNA sequencing was carried out at the Institute of Microbiology and Epidemiology (Academy of Military Medical Sciences, Beijing, China) using the Ion Torrent Personal Genome Machine (PGM)[5]. Sequence reads were assembled using the Newbler v2.9 software package (454 Life Sciences, Branford, CT).

Analysis of PaoP5 genome termini. The software package DNAStar[6] was used to simulate the restriction enzyme mapping of the PaoP5 genome sequence. The PaoP5 DNA was digested by restriction endonuclease SpeI (NEB, MA, USA). In a reaction system of 20 μL, 10 units of the restriction endonuclease and 200 ng of PaoP5 DNA were used. The mixture was incubated at 37 ℃ for 120 min and then used to perform agarose gel electrophoresis. The sequencing coverage plot of the

PaoP5 genome was generated by the Newbler v2.9 software package (454 Life Sciences, Branford, CT). Phage DTR sequences were subjected to BlastN analysis by using EasyFig (http://mjsull.github.io/Easyfig/)[7].

Sequence analysis and genome annotation. The software package DNAStar[6] and DNAMAN (http://www.lynnon.com/) were used to analyze the general features of the PaoP5 genome sequence. PaoP5 genes were predicted using RAST (http://rast.nmpdr.org/)[8] and fgenesV (http://linux1.softberry.com/berry.phtml?topic=virus&group=programs&subgroup=gfindv), and the results were merged and checked manually. DNA sequences and protein sequences were scanned for homologs via BLAST[9]. The circular presentation of the PaoP5 genome was performed using BLAST Ring Image Generator (BRIG) (http://brig.sourceforge.net/)[10] and CGView (http://stothard.afns.ualberta.ca/cgview_server/)[11], and the results of BRIG and CGView were combined to present the genome map.

References

1 Sambrook, J. & Russell, D. W. *The condensed protocols from Molecular cloning : a laboratory manual*. (Cold Spring Harbor Laboratory Press, 2006).

2 Govind, R., Fralick, J. A. & Rolfe, R. D. Genomic organization and molecular characterization of Clostridium difficile bacteriophage PhiCD119. *Journal of bacteriology* **188**, 2568-2577, doi:10.1128/JB.188.7.2568-2577.2006 (2006).

3 Casas, V. & Rohwer, F. Phage metagenomics. *Methods in enzymology* **421**, 259-268, doi:10.1016/S0076-6879(06)21020-6 (2007).

4 Lu, S. G. *et al.* Genomic and Proteomic Analyses of the Terminally Redundant Genome of the Pseudomonas aeruginosa Phage PaP1: Establishment of Genus PaP1-Like Phages. *PLoS One* **8**, doi:ARTN e62933

10.1371/journal.pone.0062933 (2013).

5 Rothberg, J. M. *et al.* An integrated semiconductor device enabling non-optical genome sequencing. *Nature* **475**, 348-352, doi:10.1038/nature10242 (2011).

6 Rosseel, T. *et al.* DNase SISPA-next generation sequencing confirms Schmallenberg virus in Belgian field samples and identifies genetic variation in Europe. *PLoS One* **7**, e41967, doi:10.1371/journal.pone.0041967 (2012).

7 Sullivan, M. J., Petty, N. K. & Beatson, S. A. Easyfig: a genome comparison visualizer. *Bioinformatics* **27**, 1009-1010, doi:10.1093/bioinformatics/btr039 (2011).

8 Brettin, T. *et al.* RASTtk: a modular and extensible implementation of the RAST algorithm for building custom annotation pipelines and annotating batches of genomes. *Scientific reports* **5**, 8365, doi:10.1038/srep08365 (2015).

9 Singh, H. & Raghava, G. P. S. BLAST-based structural annotation of protein residues using Protein Data Bank. *Biol Direct* **11**, doi:Artn 4
10.1186/S13062-016-0106-9 (2016).

10 Alikhan, N. F., Petty, N. K., Ben Zakour, N. L. & Beatson, S. A. BLAST Ring Image Generator (BRIG): simple prokaryote genome comparisons. *BMC genomics* **12**, 402, doi:10.1186/1471-2164-12-402 (2011).

11 Grant, J. R. & Stothard, P. The CGView Server: a comparative genomics tool for circular genomes. *Nucleic acids research* **36**, W181-W184, doi:10.1093/nar/gkn179 (2008).
